# Socioeconomic disparities and difficulties to access to healthcare services among Canadian children with neurodevelopmental disorders and disabilities

**DOI:** 10.4178/epih.e2018010

**Published:** 2018-03-29

**Authors:** Sana Raouafi, Sofiane Achiche, Maxime Raison

**Affiliations:** 1 Institute of Biomedical Engineering, Polytechnic School of Montréal, Montreal, Canada; 2 Department of Mechanical Engineering, Machine Design Section, Polytechnic School of Montréal, Montreal, Canada

**Keywords:** Neurodevelopmental disorders, Children with disabilities, Socioeconomic status, Inequalities

## Abstract

**OBJECTIVES:**

The aims of this study were to identify the associations of levels of severity of neurodevelopmental disorders and disabilities (NDD/D) in children with their household socioeconomic status (SES) and their frequency of visits to a healthcare provider, and to examine how the severity of disability varied with these determinants among NDD/D subgroups, in order to inform possible social policy changes and to improve access to the healthcare system.

**METHODS:**

Data from the 2006 Participation and Activity Limitation Survey on children aged 5-14 years, collected by Statistics Canada, were analyzed (n=7,072 and weighted n=340,340). Children with NDD/D constituted those with impairments in motor, speech, neurosensory, and psychological functioning, as well as those who had issues with learning/cognition and social interactions. The weighted sample size for this group was n=111,630 (total sample size for children with limitations: n=174,810). We used logistic regression to assess the associations of household SES and frequency of visits to a healthcare provider with disability level. We included NDD/D subgroups as interaction terms in the model. Multiple correspondence analysis (MCA) was conducted to develop a profile of disability level.

**RESULTS:**

After-tax low income, family assistance, out-of-pocket expenses, needing but not receiving health services from a social worker, condition of the dwelling, and residential location were associated with the severity of NDD/D. Using MCA, 2 disability profiles could be identified based on access to healthcare, household income status, and condition of the dwelling.

**CONCLUSIONS:**

More social interventions are needed to reduce difficulties in accessing healthcare and to diminish the socially determined health inequalities faced by children with NDD/D.

## INTRODUCTION

Neurodevelopmental disorders and disabilities (NDD/D) are a group of disorders that manifest early in a child’s development. These disabilities are characterized by deficits in development that result in neurological, cognitive, behavioural, social, academic, and occupational functioning. Roughly 5% of Canadian children have a disability, and 74% of these disabilities are classified as NDD/D [1]. Over the past half century, the number of people with disabling chronic conditions has increased [2], representing a major public health concern. Some factors associated with the increased prevalence of developmental disabilities are the increased prevalence of preterm birth, infertility treatments, and lack of access to the healthcare system and health insurance coverage [3]. NDD/D can have a lifelong effect on a child’s physical, emotional, social, psychosocial, and academic functioning. The World Report on Disability [4] identified childhood disability as strongly associated with socioeconomic disadvantages (personal and environmental conditions). Inequalities in children’s socioeconomic status (SES), environmental factors, and access to healthcare are well documented [5-7]. However, the role of these inequalities in the developmental trajectories of children with NDD/D is not well known.

We hypothesize that exposure to an adverse social environment, low SES, and lack of access to quality healthcare are factors that may be associated with the severity of NDD/D. Numerous studies have shown that childhood disability is related to disadvantaged circumstances [5,7-9]. For example, Blackburn et al. [8] reported that the household income in households with a disabled child was 13% lower than in households with non-disabled children. Previous studies have shown that people who lived in rural areas experienced worse health and exhibited more health risk behaviours than those who lived in urban areas [10,11]. Beresford & Rhodes [12] suggested that children with disabilities were more likely to live in unsuitable and poor housing than their non-disabled peers. Beyond these factors, the high costs of medical services and inadequate insurance coverage are factors that impact access to the healthcare system. Newacheck & McManus [13] showed that out-of-pocket expenses were 2-3 times higher on average for disabled children than for other children. In this study, we hypothesized that factors such as socioeconomic disadvantages, socioenvironmental exposures, and access to healthcare would be different for children with different disabilities. A better understanding of the relationship between these changeable factors and the severity of disability is needed to inform health service providers so they can establish prevention strategies for the affected populations and reduce the health burden on children with NDD/D and their families.

In this research study, data from the Participation and Activity Limitation Survey (PALS) were used (1) to examine the relationships of SES, environmental exposures, and access to healthcare indicators with the level of disability in children with NDD/D; and (2) to explore how the severity of disability varied with these determinants among NDD/D subgroups.

## MATERIALS AND METHODS

### Participants

The PALS is a cross-sectional population-based study conducted in the 10 provinces and 3 territories of Canada. The sampling stratum was defined to obtain a profile of individuals with disabilities whose everyday activities are limited because of a health-related condition or problem, considering the enumeration area, age group, and severity of disability. The objective of the PALS was to provide information about children’s characteristics, including age, sex, residence, schooling, socioeconomic details, human aids, medication, difficulties and barriers to healthcare services, and type and severity of disability. Based on the PALS, the total size of the census sample for children with limitations aged 5-14 years was 174,810. The respondents targeted were parents or guardians of a child who answered affirmatively to 2 filtering questions: (1) does the child experience difficulties with hearing, seeing, moving, communicating, learning, or doing other activities; and (2) does the child have a health condition that reduces the child’s ability to participate in various activities. From the census sample, telephone interviews with 7,072 parents were conducted and their self-reports were utilised in this analysis. The development process of the 2006 PALS is described in a technical and methodological report [14]. Among children aged 5-14 years, we limited our analysis to those with NDD/D. Children with NDD/D constitute those with impairments in motor, speech, neurosensory, and psychological functioning, as well as those who have issues with learning/cognition and social interactions. Assignment into 1, 2, or 3 NDD/D subgroups has been described in a previous work by Mâsse et al. [15]. The weighted sample size for this group was n= 111,630. The sampling weights were derived by Statistics Canada [14] and adjusted for patterns of non-response and other child characteristics (age, sex, severity of disability, and province of residence). A proposal of the study to gain access to microdata files in the Research Data Centres at the University of Montreal that presented the objectives and variables to be analysed was accepted by the Social Sciences and Humanities Research Council.

### Measures

#### Socioeconomic status and environmental exposures

In the present study, indicators of SES and environmental exposure included (1) residential location (rural or urban); (2) need for familial assistance to help parents with everyday activities; (3) the total income of the census family (dichotomized at the median split of income in 2005 Canadian dollar [C$]66,343); (4) after-tax low income (yes or no); and (5) condition of the dwelling (whether the dwelling was in need of regular maintenance, minor repairs, or major repairs).

#### Health service indicators

Health service indicators help assess an individual’s access to the healthcare system. These indicators were determined by asking respondents about the following: (1) an estimate of out-of-pocket expenses (a set of options was given: less than C$200, C$200 to less than C$500, C$500 to less than C$1,000, C$1,000 to less than C$2,000, and C$2,000 or more); (2) whether there were out-of-pocket costs that were paid but were not reimbursed by a health insurance company; (3) whether there were health services that were needed by the child but not received during the past 12 months in general; and (4) the type of health services needed by the child that were not received (medical specialist, speech therapist, psychologist, or psychotherapist).

A child’s barriers to healthcare access were identified as (1) not having a health insurance card; and (2) healthcare services being considered too expensive. The format of the questionnaires was ‘yes or no’ or multiple-choice options.

#### Frequency of visits to a healthcare provider

Children with NDD/D require more visits to pediatric and other medical specialist services than their non-disabled peers. The number of visits to a healthcare professional made in the past year was determined by asking the respondent about the total number of visits made to (1) a speech therapist; (2) a psychologist; (3) an occupational therapist; and (4) a social worker. All questions were asked with 4 possible response options (at least once a week, at least once a month, less than once per month, and never).

### Statistical analysis

The chi-square and Fisher exact tests for categorical variables were used to compare socioeconomic and clinical variables according to the degree of severity. Due to the large sample size, the Cramer V was used to detect relationships that were strong enough to be practically meaningful [16]. The values from this test range from 0 to 1, with larger values of V indicating stronger associations in the variables.

Logistic regression was carried out to assess the relationships between the degree of severity as a dependent variable and socioeconomic variables, access to health services indicators, and frequency of visits to a healthcare provider as independent variables.

Questions related to access to healthcare were recorded as ‘yes,’ ‘no,’ ‘not asked,’ and ‘missing’ for respondents who did not know or refused to answer. Only parents or guardians of the child who responded either ‘yes’ or ‘no’ were included in the analysis, with a total weighted sample of n= 19,640 individuals (compared to the initial total weighted sample of n= 111,630). Degree of severity was an index variable assessing the overall level of disability (mild to moderate or severe to very severe) in NDD/D subgroups (motor, speech, neurosensory, and psychological functioning, as well as issues with learning/cognition and social interactions). To determine the severity of disability, a standardized score was calculated based on the maximum score according to the intensity and frequency of each limitation across 9 domains: hearing, seeing, motor function, speech, dexterity, learning, psychological disabilities, developmental disabilities, and issues with chronic conditions. The overall degree of severity was then calculated by averaging all standardized severity scores calculated for each type of disability. Four classes (mild, moderate, severe, and very severe) were created based on a cutoff point in the global score of the 70th percentile and close to a score of 1/8 for the children. Since these scores corresponded to someone with a maximum score for 1 type of disability, it was decided to subdivide the scale into 4 parts with 2 cutoff points: the first cutoff point was equivalent to half of the maximum score and the second cutoff point was equivalent to double the maximum score obtained for a given disability. In the current study, 2 severity classes were considered: mild to moderate and severe to very severe, because when performing cross-tabulations, the unweighted counts of some cells were less than 10, which did not meet Statistics Canada’s data release requirements. Further details regarding how severity of disability was derived can be found in the technical and methodological report [4]. Sex and age group (5-7, 8-11, or 12-14 years) were also included in the model. Because we wanted to compare the effects for different subgroups, we analyzed interaction effects between each subgroup variable, socioeconomic factors, and access to healthcare indicators. Statistical tests used an alpha of 0.05 as the level of significance. Odds ratios (ORs) were estimated with the logistic regression model for all parameters except for the interaction terms.

To identify socioeconomic patterns and disparities in use and access to healthcare among children with NDD/D with different levels of severity, multiple correspondence analysis (MCA) [17] was used. This method is part of a family of descriptive methods (clustering, principal component analysis, and multiple factor analysis) used for modeling a matrix as points in a multidimensional plane, when the data collected is categorical. Generally, this method is used in epidemiological, clinical, and social studies [18]. From the categorical variables, we constructed a disjunctive table (Burt table), the columns of which corresponded to modalities of the variables and the rows of which corresponded to individuals. MCA converts this matrix of data into a particular type of graphical display known as factor planes. Similar individuals and modalities shared by these individuals are depicted as points (in the same group) in the factor planes, and dissimilarity results in distance. This analysis enables the visualization of independent clusters on a 2-dimensional plane and permits a geometrical representation of all the information. The contribution in percentage points, from the most to the less explicative, to the construction of each axis is shown for each modality. In this research, the column points corresponded to our socioeconomic parameters and health service indicators and the row points corresponded to our observations. Only the most representative factor plane according to the total inertia explained is presented in the following analysis.

Data analysis was performed using SPSS version 24 (IBM Corp., Armonk, NY, USA). Data were rounded to the nearest digit to comply with Statistics Canada data disclosure guidelines.

## RESULTS

In the survey population, 43.5% of the children were aged between 8 and 11. Most of the children included in the sample were born in Canada (95.7%). We noted a male predominance (69.0%) among children with NDD/D. Psychological problems accounted for the most frequent subgroup (45.7%), and speech/language the least frequent (6.7%). In the motor and social groups, most children were classified as having severe to very severe overall disability; the percentages for severe to very severe vs. mild to moderate were 68.9 vs. 31.1% and 82.3 vs. 17.8% for the motor and social groups, respectively. Table 1 summarizes the demographic and descriptive information of children with NDD/D. Table 2 reports the levels of mild to moderate and severe to very severe disabilities in children with NDD/D by socioeconomic characteristics. Of the weighted sample (n= 111,630) of children with NDD/D aged 5 to 14, 45.8% experienced a mild to moderate disability and 54.2% a severe to very severe disability. Residential location, census family total income, after-tax low income, family assistance, and condition of the dwelling were significantly associated with the level of disability (p< 0.001). All the relationships were found to be weak using the Cramer V, except for the association between levels of disability and family assistance (Cramer V= 0.386). Out-of-pocket expenses, estimated out-of-pocket expenses, and health services needed but not received from a specialist medical doctor, speech therapist, or psychotherapist were significantly associated with the level of disability (p< 0.001). All the relationships were fairly strong according to the Cramer V (varying between 0.258 and 0.261) except for the associations between the level of disability and out-of-pocket expenses and estimated out-of-pocket expenses (Table 3). Table 4 presents the association between the level of severity and the frequency of visits to a healthcare provider in the past 12 months. A statistically significant relationship was found between the level of disability and the frequency of visits to a speech therapist, psychologist, occupational therapist, or social worker. All the relationships were moderate to strong, with the Cramer V varying between 0.204 and 0.304, except for the association between the level of disability and frequency of visits to a psychologist.

**Figure 1.**
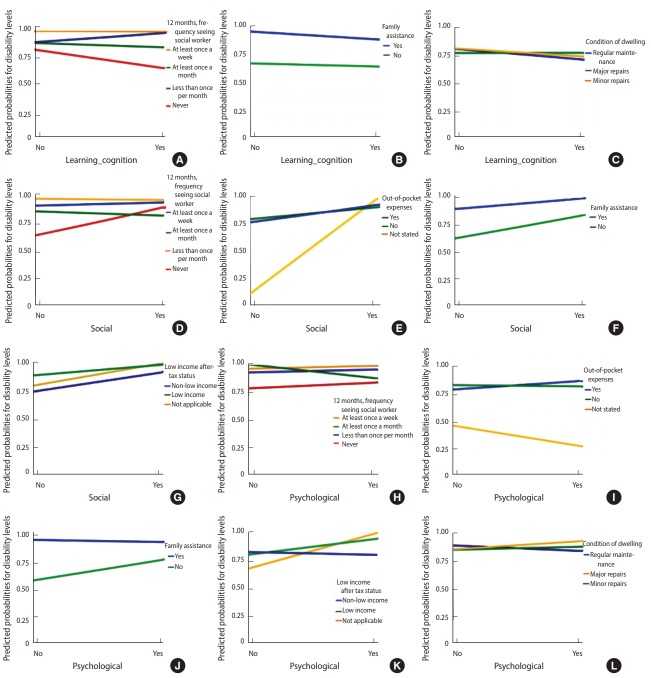
Predicted probabilities of disability levels. (A) Learning/cognition×In the past 12 months, frequency of seeing a social worker. (B) Learning/cognition×Family assistance. (C) Learning/cognition×Condition of dwelling. (D) Social×In the past 12 months, frequency of seeing a social worker. (E) Social×Out-of-pocket expenses. (F) Social×Family assistance. (G) Social×Low income status. (H) Psychological×In the past 12 months, frequency of seeing a social worker. (I) Psychological×Out-of-pocket expenses. (J) Psychological×Family assistance. (K) Psychological×Low income status. (L) Psychological×Condition of dwelling.

Logistic regression was performed on uncorrelated variables to identify the best predictors of the level of severity. Multicollinearity was detected between the following parameters: between census family total income and after-tax low income; between frequency of visits to a speech therapist, psychologist, occupational therapist, and social worker; and between parameters related to access to health services. In cases where variables were highly correlated, as measured by the Cramer V (> 0.90), only the variable with the strongest association with the degree of severity was entered into the logistic regression model to avoid problems with collinearity. Six variables were then considered: residential location, condition of the dwelling, family assistance, frequency of visits to a social worker, out-of-pocket expenses and after-tax low income. The characteristics of age and sex were also included in the model. To test whether the relationship between severity of disability and SES varied by NDD/D subgroups, the severity of disability was regressed onto the SES and NDD/D subgroups and the interaction terms for NDD/D subgroups and SES characteristics simultaneously. Answers of ‘no’ served as the reference group for all NDD/D subgroups. Motor impairments were excluded from the analysis due to a numerical problem created by the presence of cell values with small frequencies. Logistic regression analysis revealed that 55.5% (Nagelkerke R-square) of the variance in levels of severity was explained by SES, NDD/D subgroups, and the interaction terms for NDD/D subgroups and SES characteristics simultaneously (Table 5). Children aged between 8 and 11 were more likely to report severe to very severe disabilities than younger children (OR, 1.56). The OR of having severe disabilities was 2.07 for females compared to males. Logistic regression showed that the features significantly associated with level of disability were residential location (urban: OR, 2.75; 95% confidence interval [CI], 2.07 to 3.64), condition of the dwelling (major repairs: OR, 3.09; 95% CI, 2.05 to 4.67; minor repairs: OR, 1.77; 95% CI, 1.30 to 2.40), family assistance (no: OR, 0.02; 95% CI, 0.01 to 0.03), out-of-pocket expenses (no: OR, 5.15; 95% CI, 3.75 to 7.06), aftertax low income (OR, 0.22; 95% CI, 0.12 to 0.38), frequency of visits to a social worker (never: OR, 0.43; 95% CI, 0.30 to 0.62), social interaction (yes: OR, 329.06; 95% CI, 136.30 to 794.40), sensory impairments (yes: OR, 1.35; 95% CI, 1.04 to 1.74) and psychological impairments (yes: OR, 0.31; 95% CI, 0.21 to 0.47). Figure 1 and Supplementary Material 1 present the associations between levels of severity regarding the interaction terms for NDD/ D subgroups and SES characteristics simultaneously. The risk of having a child with severe disability was higher among children with speech-language impairments whose families had out-of-pocket expenses but less among children with speech-language impairments whose families did not have out-of-pocket expenses. With the presence of learning/cognition impairments, the risk of having a child with a severe disability was higher among families; those who needed help with housework, family, and personal activities (vs. families who do not need family assistance); those who lived in a household needing major or minor repairs (vs. families who lived in a household needing regular maintenance); and those whose children visited a social worker at least once a week (vs. those who never visited a social worker). For children with social impairments, the predicted probability of having a severe disability was significantly lower among families who did not report out-of-pocket expenses (vs. those with out-of-pocket expenses), higher for children whose families needed help (vs. those who did not need family assistance), higher among children with after-tax low income (vs. children without after-tax low income), lower among children who lived in a household needing major or minor repairs (vs. children who lived in a household needing regular maintenance), lower for children who lived in urban areas (vs. rural areas), and higher among children who visited a social worker less than once per month. For children with psychological impairments, the predicted probability of having a severe disability was significantly higher among families who reported out-of-pocket expenses (vs. those with no out-of-pocket expenses), who needed help with housework, family, and personal activities (vs. those who did not need help), with after-tax low income (vs. families without after-tax low income), who lived in a household needing major or minor repairs (vs. those who lived in a household needing regular maintenance), and whose children visited a social worker at least once a week (vs. never). No other interaction effects were significant, and hence are not reported.

**Figure 2.**
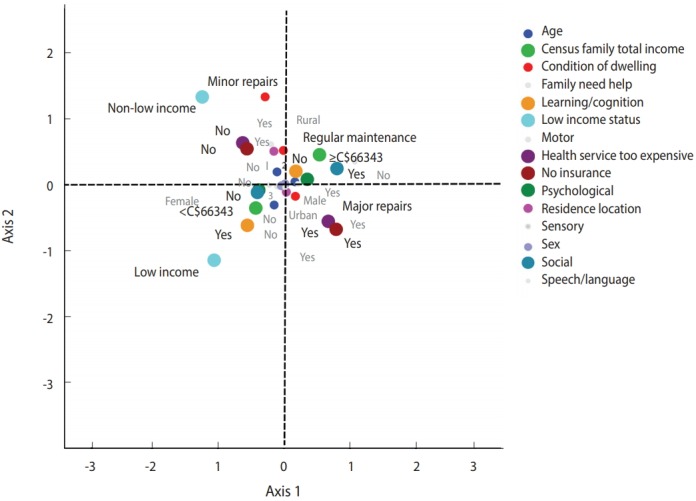
The column points of the first factor plane of the multiple correspondence analysis (axes 1 and 2).

When MCA was applied to the weighted sample (n = 19,640 participants) using age; sex; census family total income; after-tax low income; family assistance; residential location; condition of the dwelling; lack of health insurance; inability to afford healthcare services; and difficulties in motor, speech/language, learning/ cognition, social interaction, neurosensory, and psychological functioning as imputed values, it was found that the total inertia explained by the first factor plane (Figure 2) was equal to 29.8%. Supplementary Material 2 presents the main parameters that best contributed to creating the first and second axes. Figure 2 presents the 2-dimensional map of MCA with the coordinates of n= 19,640 weighted respondents. The first dimension (eigenvalue= 2.917), explained 16.4% of the total inertia. The negative pole of the first axis includes children who lived in a household with an income of less than C$66,343 per year (after-tax low income) but did not have social problems. At the positive pole, dimension 1 encompasses children who lived in a household with an income of more than C$66,343 per year (not after-tax low income) but who had social problems. The second dimension (eigen value= 1.754), explained 13.5% of the total inertia. The most discriminant parameters were “health services (that were) too expensive” and “health services not covered by insurance.” In this bipolar dimension, the negative pole includes children who lived in a dwelling needing major repairs, children who had learning/cognition problems, children who had difficulty accessing healthcare services because they were too expensive, and children who did not have a healthcare insurance card. The positive pole includes children who were more likely to live in a dwelling needing minor repairs or regular maintenance, less likely to report learning/cognition problems, and less likely to have difficulty accessing healthcare services. Based on the distribution of the individuals in the quadrants of the factor plane, 2 profiles were identified. The right-bottom and the left-bottom quadrants present the first profile, which represents children who lived in a dwelling needing major repairs, who had learning/cognition problems, who had difficulty accessing healthcare services because they were too expensive, who did not have a healthcare insurance card, and who lived in a household with an income of less than C$66,343 per year (i.e., with low income status), but who did not have social problems. The right-top and the left-top quadrants represent children who lived in a household with an income of more than C$66,343 per year (not low income status), had social problems, lived in a dwelling needing minor repairs or regular maintenance, did not have learning/ cognition problems, and did not have difficulty accessing healthcare services.

## DISCUSSION

This study was designed to identify which socioeconomic parameters and variables describing access to healthcare indicators were the most pertinent for developing a profile of disability severity among Canadian children aged 5-14 years with NDD/D.

The main result, reported in Tables 2-4, was the strong association between severity of disability and socioeconomic disadvantages including low income status, family assistance, out-of-pocket expenses, and needing but not receiving health services from a social worker. Our results illustrate that the relationships of socioeconomic parameters and healthcare indicators with severity of disability are best understood in concert, rather than separately. These differences were apparent only in certain NDD/D subgroups. As shown in Figure 1, the traditional relationship of lower SES and difficulty accessing healthcare services with poorer health and severe disability emerged among children with speech/language, learning/cognition, social, and psychological impairments.

These findings are consistent with results from other studies in which disability was related to socioeconomic gradients [6,7,12,19, 20]. Our MCA showed that low income status, condition of the dwelling, and healthcare indicators (high medical costs or not having insurance coverage) were the parameters that contributed most to the disability profile of children with NDD/D (Figure 2 and Supplementary Material 2). Both our findings and those of previous studies indicate that disability in children is socially patterned. Most studies comparing children’s disabilities in rural and urban areas have reported that children who lived in rural areas experienced worse health and exhibited more health risk behaviours than those who lived in urban areas [10,11]. These results are in concordance with our findings, specifically for children with social interaction impairments (Figure 1). Our results indicate that severely disabled children (specifically, those with learning/ cognition and psychological impairments) were more likely to live in dwellings needing major or minor repairs than children with mild to moderate disabilities. In the UK, an analysis of the characteristics and circumstances of disabled children found that poor or unsuitable housing was correlated with childhood disability [8]. For children with learning/cognition, social, and psychological impairments, the predicted probability of having a severe disability was significantly higher among families who needed help with housework, family, and personal activities. Out-of-pocket expenses influenced the level of disability in speech/language, social, and psychological impairments; in particular, the risk of severe disability, as depicted in Figure 1, was much higher for children with speech/language, social, and psychological impairments who had out-of-pocket expenses. Stabile & Allin [21], found that out-of-pocket expenses were higher in families with disabled children than in families that did not have disabled children, especially those with special needs.

The association between SES and health is well documented in the literature [22]. Although many risk factors have been identified, their consequences on the developmental trajectories of childhood are poorly understood. The present study adds several new insights, as 3 of our findings deserve additional discussion.

First, uninsured children and children for whom medical services were too expensive were more likely to have a severe to very severe disability. There is evidence that the costs of disability are significant [4], but excess costs of medical services are also probably due to a lack of health insurance coverage and poor health status [23]. This suggests that insurance coverage is related to access to care for children with disabilities [24], and consequently that a lack of insurance coverage is associated with worse health outcomes and increased severity of disability.

Second, we found that children with a severe disability were more likely to live in low income households and dwellings needing repairs. Families of children with a disability have been found to be more likely to live in unsuitable homes and more likely to have poverty-level incomes than families with no disabled children [8,10-12]. Given the income disparities that we found with MCA for disability profiling, our results indicate that children who had a severe disability and social or psychological impairments were more likely both to be from low income households and to have difficulty accessing healthcare services than children who had a less severe disability [25].

Finally, we found that children with a severe to very severe disability were more likely to report social impairments (Table 5). Moreover, our MCA showed that children with a severe disability were more likely to report learning/cognition impairments. This result is not surprising since this limitation was the most common type of disability reported for children aged 5 to 14 [14]. Additionally, social interaction impairments are common behavioural characteristics of individuals with learning disabilities [26,27].

This research study has the following notable strengths. First, it included a nationally representative sample of Canadian children, in contrast to most previous qualitative studies, which included fewer participants to identify relationships between health experiences of disabled children and socioeconomic factors. Second, specific types of NDD/D were included, with both mild to moderate and severe to very severe levels of disability, which contributed to the diversity of the sample.

In a cross-sectional study, the data for risk factors and outcome are simultaneously obtained, so it is difficult to interpret any causal/directional relationship. While the primary outcome variable was disability levels, and the predictor variables for this study were exposure to socioeconomic disadvantages, poor housing, and difficulty accessing healthcare services, this relationship could have been reversed, but the directionality of the relationship could not be investigated. Another limitation of this study is that the PALS contained no information about family structure, parental employment, and education, which may be important factors related to the severity of disability of children with NDD/D. In addition, a recent study showed that the use and frequency of use of assistive mobility devices may impact the severity of disability [28]. Further studies will be needed to validate the proposed analysis including these factors.

In conclusion, exposure to socioeconomic disadvantages, poor housing, and difficulty accessing healthcare services were associated with greater severity of disability among children with NDD/ D. The World Report on Disability [4], makes some recommendations to ensure healthcare equity and to promote a focus on determinants of health of people with disabilities, especially on their living conditions. Some such initiatives have been already implemented in many countries. However, to improve the quality of these interventions and to reduce the health burden of children with NDD/D, more efforts are needed to provide more robust and comprehensive data on disability and to characterize in detail the impact of medical, environmental, and social factors.

## Figures and Tables

**Table 1.** Demographic and descriptive information of children (5-14 years) with NDD/D in PALS

	Total (weighted n=111,630, %)
Age (yr)	
5-7	23.5
8-11	43.5
12-14	33.0
Sex	
Male	69.0
Female	31.0
Place of birth	
Born in Canada	95.7
Born outside Canada	4.3
NDD/D subgroups	
Motor	9.8
Speech/language	6.7
Learning/cognition	25.1
Social	18.5
Sensory	15.1
Psychological	45.7
Severity of overall disability by NDD/D subgroups	
Motor	
Mild to moderate	31.1
Severe to very severe	68.9
Speech/language	
Mild to moderate	42.4
Severe to very severe	57.6
Learning/cognition	
Mild to moderate	46.8
Severe to very severe	53.2
Social	
Mild to moderate	17.8
Severe to very severe	82.3
Sensory	
Mild to moderate	67.8
Severe to very severe	32.2
Psychological	
Mild to moderate	46.3
Severe to very severe	53.7

NDD/D, neurodevelopmental disorders and disabilities; PALS, Participation and Activity Limitation Survey 2006.

**Table 2.** Socioeconomic characteristics by the overall degree of disability of children with NDD/D in PALS

	Total (weighted n=111,630)			
	Mild to moderate (n=51,180, %)	Severe to very severe (n=60,450, %)	Cramer’s V	p-value
Residential location				
Rural	80.3	82.1	0.023	<0.001
Urban	19.7	17.9		
Census family total income (Canadian dollar)				
<66,343	52.2	61.4	0.093	<0.001
≥66,343	47.8	38.6		
Low after-tax income status				
Non-low income	86.5	78.0	0.111	<0.001
Low income	13.5	22.0		
Family assistance				
Yes	10.0	45.2	0.386	<0.001
No	90.0	54.9		
Condition of dwelling				
Regular maintenance	50.7	50.9	0.038	<0.001
Major repairs	12.8	15.0		
Minor repairs	36.5	34.1		

NDD/D, neurodevelopmental disorders and disabilities; PALS, Participation and Activity Limitation Survey 2006.

**Table 3.** Access to healthcare indicators

	Total (weighted n=111,630)			
	Mild to moderate (n=51,180, %)	Severe to very severe (n=60,450, %)	Cramer’s V	p-value
Out-of-pocket expenses				
Yes	21.3	30.1	0.163	<0.001
No	69.5	67.3		
Not stated	9.2	2.6		
Estimate of out-of-pocket expenses (Canadian dollar)				
<200	24.4	13.4	0.136	<0.001
200-500	24.5	20.5		
500-1,000	25.1	27.2		
1,000-2,000	13.1	13.8		
≥2,000	13.1	25.1		
Needed but did not receive health service				
Yes	7.0	26.7	0.261	<0.001
No	92.2	71.9		
Not asked	0.9	1.3		
Needed a specialist medical doctor				
Yes	0.6	2.7	0.258	<0.001
No	6.4	24.0		
Not asked	93.0	73.3		
Needed a speech therapist				
Yes	2.2	9.2	0.258	<0.001
No	4.8	17.6		
Not asked	93.0	73.3		
Needed a psychologist or a psychotherapist				
Yes	1.3	5.8	0.258	<0.001
No	5.6	20.9		
Not asked	93.0	73.3		

**Table 4.** Frequency of visits to a healthcare provider in the past 12 months

	Total (weighted n=111,630)			
	Mild to moderate (n=51,180, %)	Severe to very severe (n=60,450, %)	Cramer’s V	p-value
Speech therapist				
At least once a week	8.1	17.8	0.204	<0.001
At least once a month	5.5	9.2		
Less than once per month	7.2	12.5		
Never	77.2	59.2		
Not stated	2.1	1.3		
Psychologist				
At least once a week	2.6	4.5	0.177	<0.001
At least once a month	4.0	8.2		
Less than once per month	16.4	26.8		
Never	75.6	59.1		
Not stated	1.3	1.3		
Occupational therapist				
At least once a week	1.2	6.8	0.273	<0.001
At least once a month	3.9	11.4		
Less than once per month	6.9	16.8		
Never	86.8	63.2		
Not stated	1.1	1.9		
Social worker				
At least once a week	1.4	5.8	0.304	<0.001
At least once a month	5.6	12.0		
Less than once per month	7.9	24.1		
Never	81.9	57.3		
Not stated	3.2	0.8		

**Table 5.** Associations between socioeconomic characteristics, frequency of visits to a healthcare provider and overall degree of disability of children with NDD/D in PALS: multivariate logistic regression model

	Total (weighted n=111,630)
	aOR (95% CI)
Age (yr)	
5-7	1.00 (reference)
8-11	1.56 (1.36, 1.80)^***^
12-14	0.76 (0.66, 0.88)^***^
Residential location	
Rural	1.00 (reference)
Urban	2.75 (2.07, 3.64)^***^
Condition of dwelling	
Regular maintenance	1.00 (reference)
Major repairs	3.09 (2.05, 4.67)^***^
Minor repairs	1.77 (1.30, 2.40)^***^
Family assistance	
Yes	1.00 (reference)
No	0.02 (0.01, 0.03)^***^
Sex	
Male	1.00 (reference)
Female	2.07 (1.89, 2.36)^***^
In past 12 months, frequency of seeing a social worker	
At least once a week	1.00 (reference)
At least once a month	-
Less than once per month	-
Never	0.43 (0.30, 0.62)^***^
Out-of-pocket expenses not reimbursed from health professional	
Yes	1.00 (reference)
No	5.15 (3.75, 7.06)^***^
Not stated	-
Low after-tax income status	
Yes	0.22 (0.12, 0.38)^***^
No	1.00 (reference)
Not stated	-
NDD/D subgroups	
Speech/language (yes)	-
Learning/cognition (yes)	-
Social (yes)	329.06 (136.30, 794.40)^***^
Sensory (yes)	1.35 (1.04, 1.74)^*^
Psychological (yes)	0.31 (0.21, 0.47)^***^

NDD/D, neurodevelopmental disorders and disabilities; PALS, Participation and Activity Limitation Survey 2006; aOR, adjusted odds ratio; CI, confidence interval.

* p<0.05,

*** p<0.001.

## SUPPLEMENTARY MATERIALS

Supplementary Material 1: Table S1 is available at http://www.e-epih.org/.

Supplementary Material 2: Table S2 is available at http://www.e-epih.org/.
