# Supplementary material for: Socioeconomic disparities and difficulties to access to healthcare services among Canadian children with neurodevelopmental disorders and disabilities
Source: Epidemiol Health. 2018 Mar 29;40:e2018010. doi: 10.4178/epih.e2018010 (PMC6004430; doi:10.4178/epih.e2018010)
Supplement: Supplementary file 1 [file epih-40-e2018010-supplementary.pdf]

Table S1. Associations between levels of disability and the interaction terms for NDD/D subgroups in PALS and SES characteristics simultaneously: adjusted ORs and 95% CIs from multivariate logistic regression model

	Total (weighted n=111,630) %	
	**OR (95% CI)**	**p-value**
**Speech/language * Out-of-pocket expenses (yes)**		<0.001
No	0.004 (0.002, 0.008)	<0.001
Not stated	-	0.990
**Learning/cognition * In past 12 months, frequency of seeing a social worker (never)**		<0.001
At least once a week	-	0.992
A least once a month	-	0.992
Less than once per month	75.86 (38.27, 150.37)	<0.001
**Learning/Cognition * Family assistance (yes)**	-	<0.001
No	3.48 (2.37, 5.13)	<0.001
**Learning/Cognition * Condition of dwelling (regular maintenance)**		<0.001
Major repairs	0.21 (0.13, 0.33)	<0.001
Minor repairs	0.27 (0.19, 0.38)	<0.001
**Social* In past 12 months, frequency of seeing a social worker (never)**		<0.001
At least once a week	-	0.996
At least once a month	0.02 (0.01, 0.05)	<0.001
Less than once per month	5.30 (3.33, 8.40)	<0.001
**Social * Out-of-pocket expenses (yes)**	-	<0.001
No	0.28 (0.19, 0.41)	<0.001
Not stated	-	0.989
**Social * Family assistance (yes)**	-	<0.001
No	0.14 (0.06, 0.31)	<0.001
**Social * Low after-tax income status (no)**	-	<0.001
Yes	615.34 (138.14, 2740.90)	<0.001
Not stated	-	1.000
**Social * Condition of dwelling (regular maintenance)**	-	<0.001
Major repairs	0.01 (0.00, 0.03)	<0.001
Minor repairs	0.09 (0.06, 0.14)	<0.001
**Social * Residential location (rural)**	-	<0.001
Urban	0.44 (0.27, 0.69)	<0.001
**Psychological * In past 12 months, frequency of seeing a social worker (never)**	-	<0.001
At least once a week	-	0.994
A least once a month	-	0.986
Less than once per month	3.08 (2.08, 4.56)	<0.001
**Psychological * Out-of-pocket expenses (yes)**	-	<0.001
No	0.23 (0.16, 0.31)	<0.001
Not stated	-	0.993
**Psychological * Family assistance (yes)**	-	<0.001

No	9.96 (6.92, 14.34)	<0.001
**Psychological * Low after-tax income status (no)**	-	<0.001
Yes	47.63 (26.66, 85.07)	<0.001
Not stated	-	0.996
**Psychological * Condition of dwelling (regular maintenance)**	-	<0.001
Major repairs	3.70 (2.43, 5.64)	<0.001
Minor repairs	2.65 (1.94, 3.63)	<0.001

NDD/D, neurodevelopmental disorders and disabilities; PALS, Participation and Activity Limitation Survey 2006; OR, odds ratio; CI, Confidence interval.

.
