# Supplementary material for: Socioeconomic disparities and difficulties to access to healthcare services among Canadian children with neurodevelopmental disorders and disabilities
Source: Epidemiol Health. 2018 Mar 29;40:e2018010. doi: 10.4178/epih.e2018010 (PMC6004430; doi:10.4178/epih.e2018010)
Supplement: Supplementary file 2 [file epih-40-e2018010-supplementary2.pdf]

Table S2. Discrimination measures

Axes	Parameters	Correlation
1	Census Family Total Income	0.231
1	Low-income status	0.221
2	Condition of dwelling	0.169
2	Learning cognition difficulties	0.124
1	Social problems	0.324
2	Health services too expensive	0.479
2	Health services not covered by insurance	0.430
